# Typhoid Fever

**Published:** 1903-04

**Authors:** 


Typhoid Fever.—By J. T. Moore, M. D., M. C. P. S., Professor of Theory and Practice of Medicine, Medical Department of Hamline University, Minneapolis, Minn. Pages 159. Price $1.00 net. G. P. Engelhard & Co., Chicago, 1902.

This is a handy and reliable little book on the subject of typhoid fever. Nothing new is attempted. The treatment is orthodox, and seems to emanate from observation at the bed-side. It is a practical brochure. T. J. B.

